# A Cytotoxic, Co-operative Interaction Between Energy Deprivation and Glutamate Release From System x_c_^−^ Mediates Aglycemic Neuronal Cell Death

**DOI:** 10.1177/1759091415614301

**Published:** 2015-11-03

**Authors:** Trista L. Thorn, Yan He, Nicole A. Jackman, Doug Lobner, James A. Hewett, Sandra J. Hewett

**Affiliations:** 1 Department of Biology, Program in Neuroscience, Syracuse University, NY, USA; 2 Department of Neuroscience and Physiology, SUNY Upstate Medical University, Syracuse, NY, USA; 3 Department of Neuroscience, University of Connecticut Health Center, Farmington, CT, USA; 4 Department of Biomedical Sciences, Marquette University, Milwaukee, WI, USA; Nicole A. Jackman is now at Department of Anesthesia & Perioperative Care, University of California San Francisco, CA

**Keywords:** aglycemia, cystine, glutamate, neuronal injury, glucose deprivation, cell culture

## Abstract

The astrocyte cystine/glutamate antiporter (system x_c_^−^) contributes substantially to the excitotoxic neuronal cell death facilitated by glucose deprivation. The purpose of this study was to determine the mechanism by which this occurred. Using pure astrocyte cultures, as well as, mixed cortical cell cultures containing both neurons and astrocytes, we found that neither an enhancement in system x_c_^−^ expression nor activity underlies the excitotoxic effects of aglycemia. In addition, using three separate bioassays, we demonstrate no change in the ability of glucose-deprived astrocytes—either cultured alone or with neurons—to remove glutamate from the extracellular space. Instead, we demonstrate that glucose-deprived cultures are 2 to 3 times more sensitive to the killing effects of glutamate or *N*-methyl-D-aspartate when compared with their glucose-containing controls. Hence, our results are consistent with the weak excitotoxic hypothesis such that a bioenergetic deficiency, which is measureable in our mixed but not astrocyte cultures, allows normally innocuous concentrations of glutamate to become excitotoxic. Adding to the burgeoning literature detailing the contribution of astrocytes to neuronal injury, we conclude that under our experimental paradigm, a cytotoxic, co-operative interaction between energy deprivation and glutamate release from astrocyte system x_c_^−^ mediates aglycemic neuronal cell death.

## Introduction

Hypoglycemia is a common and serious medical emergency that arises as a complication in patients attempting to tightly regulate glucose levels with insulin and is the limiting factor in the development of strategies that aim to maintain normoglycemia in diabetics (Lincoln et al., 1996; Lacherade et al., 2009). Hypoglycemia is also a complication in neonates and in patients with insulin-producing tumors (Anderson et al., 1967; de Herder, 2004) and occurs as a consequence of brain ischemia. Idiopathic, diabetic, and reactive hypoglycemia, (i.e., low blood glucose concentrations of <3.9 mM) can produce a variety of cognitive symptoms resulting from an inadequate supply of glucose to the brain. Owing to the fact that there is a linear relationship between blood and brain glucose levels (Choi et al., 2001), a large drop in blood glucose concentration (<2.0 mM) renders the brain aglycemic, which can result in a severe impairment of cognition, seizures, unconsciousness, and coma. If glucose levels are not restored within 30 min time, death of vulnerable cortical and hippocampal neurons also ensue (Suh et al., 2003; Auer, 2004).

Despite being initiated by glucose deprivation (GD), both *in vitro* and *in vivo* investigations showed that hypoglycemic neuronal cell death is not a direct result of energy failure but instead is mediated by glutamate excitotoxicity (Engelsen et al., 1986; Linden et al., 1987; Monyer et al., 1989; Papagapiou and Auer, 1990; Jackman et al., 2012). Adding to this understanding, we recently described system x_c_^−^—a transporter that exports L-glutamate in exchange for L-cystine (Bannai, 1984b)—in astrocytes as a source of glutamate required for the initiation of non-cell autonomous neuronal injury following GD *in vitro* (Jackman et al., 2012). The precise mechanism by which injury is facilitated by astrocyte system x_c_^−^ was not ascertained therein and thus is the focus of this study.

Maintenance of extracellular glutamate concentrations within a narrow physiological range involves control of its release as well as its uptake, both of which are efficiently managed by glutamate transporters expressed predominantly by astrocytes. Pertinently, numerous studies demonstrate that system x_c_^−^ is an important contributor to the ambient extracellular glutamate levels that bathe the central nervous system (CNS) *in vivo* (Jabaudon et al., 1999; Warr et al., 1999; Baker et al., 2002a, 2002b; Melendez et al., 2005; Augustin et al., 2007; Featherstone and Shippy, 2008; De Bundel et al., 2011; Massie et al., 2011) with maximal transporter activity estimated to theoretically increase extracellular glutamate by 0.6 µM/s (Warr et al., 1999; Cavelier et al., 2005). Conversely, nearly 90% of glutamate is removed by astrocytic Excitatory Amino Acid Transporters 1 (EAAT1) and 2 (EAAT2; Rothstein et al., 1996; Anderson and Swanson, 2000). Thus, excitotoxic processes subsequent to GD in our system could result via the enhancement of astrocytic glutamate release from system x_c_^−^, by a reduction in astrocyte cellular reuptake or by a combination of both. Hence, the overall purpose of this study was to investigate whether changes in system x_c_^−^ expression or alterations in glutamate handling following GD occur(s) and contributes to aglycemic neuronal cell death.

## Materials and Methods

### Animals

This study was conducted in accordance with the National Institutes of Health guidelines for the use of experimental animals and was approved by the Institutional Animal Care and Use Committee at both The University of Connecticut Health Center and Syracuse University. Time-pregnant CD1 mice were obtained from Charles River Laboratories (Wilmington, MA).

### Cell Culture

*Media stock (MS)*: L-glutamine-free modified Eagle’s medium (Earl’s salt; MediaTech, Herndon, VA) supplemented with L-glutamine, glucose, and sodium bicarbonate to a final concentration of 2.0, 25.7, and 28.2 mM, respectively; *Glial plating media*: MS containing 10% heat-inactivated fetal bovine serum (Hyclone, Logan, UT) and 10% heat-inactivated calf serum (CS; Hyclone, Logan, UT), 10 ng/ml epidermal growth factor (Invitrogen, Carlsbad, CA), and 50 IU penicillin/50 µg/ml streptomycin (Gibco®, Life Technologies, Grand Island, NY); *Glial/mixed culture maintenance media*: MS containing 10% CS and 50 IU penicillin/50 µg/ml streptomycin; *Neuronal plating media*: MS containing 5% CS and 5% heat-inactivated bovine growth serum (Hyclone, Logan, UT) and 50 IU penicillin/50 µg/ml streptomycin (Gibco®, Life Technologies, Grand Island, NY); *Neuronal maintenance media*: neurobasal medium (Gibco®, Life Technologies, Grand Island, NY) containing 2 mM L-glutamine, 1× B27 supplement (Gibco®, Life Technologies, Grand Island, NY), and 50 IU penicillin/50 µg/ml streptomycin (Gibco®, Life Technologies, Grand Island, NY); *HEPES-controlled salt solution (HCSS)*: 120 mM NaCl, 5.4 mM KCl, 0.8 mM MgCl_2_, 1.8 CaCl_2_, 15 mM glucose, 20 mM HEPES, 10 mM NaOH, 10 µM glycine, and 0.001% phenol red; *Glucose-free balanced salt solution* (BSS_0_): 116 mM NaCl, 5.4 mM KCl, 0.8 mM MgCl_2_, 1 mM NaH_2_PO_4_, 26.2 mM NaHCO_3_, 1.8 mM CaCl_2_, 0.01 mM glycine, and 2 mM L-glutamine. Unless otherwise indicated, BSS contained 1 × MEM amino acids (Life Technologies, Grand Island, NY).

*Primary astrocytes* were cultured from cortices of postnatal Day 1 to 3 CD1 mouse pups as described previously (Trackey et al., 2001; Uliasz et al., 2012). Cortices were dissected, pooled, and cells dissociated by trypsin digestion (0.025%, 15 min, 37℃). Cells were plated 400 µl/well in glial plating medium (two hemispheres/10 ml/plate; Falcon Primaria 24-well plates; BD Biosciences, Lincoln Park, NJ). Once confluent, astrocyte monolayers were treated with 8 µM β-D-cytosine arabinofuranoside (AraC) once for 4 to 7 days to reduce the number of microglia. Cells were then placed in maintenance media (*vide supra*), which was replaced once per week until experimentation. Purified astrocyte cultures were generated by removing residual microglia by treating monolayers with 50 to 75 mM L-leucine methyl ester for 30 to 90 min, 1 day prior to experimentation (Hamby et al., 2006; Uliasz et al., 2012). Cultures were used for experimentation at ≤35 days *in vitro*.

*Primary neuronal cultures* were derived from dissociated cortical cells of embryonic Day 15 CD1 mouse fetuses. Following dissection and dissociation by trypsin digestion (*vide supra*), cells were diluted to a concentration of 10^6^ cells/ml of neuronal plating medium and then plated into polyethyleneimine coated 24-well plates (Costar®, Corning, NY). Four hr later, the medium was exchanged to neuronal maintenance medium. Two days later, cultures were treated with 1 µM AraC once for 2 days to prevent glial cell growth. The medium was partially replenished (1/2 volume exchange) at DIV 4. Experiments were performed on purified neuronal cultures after 6 days *in vitro*.

*Mixed cortical cell cultures* containing an approximate 50:50 neuron-to-astrocyte ratio were prepared by culturing dissociated cells from embryonic Day 15 CD1 mouse fetuses on to a confluent layer of microglia-depleted astrocytes in neuronal plating media. The plating medium was partially replaced (2/3 exchange) with maintenance medium at Days 5 and 9 *in vitro*. At Day 7, cultures were treated with 8 µM of AraC once to prevent microglial cell growth*.* Two days prior to experimentation, mixed cortical cell cultures were placed into MS (*vide supra*). Experiments were performed at 14 days *in vitro*. All cultures were maintained at 37℃ in a humidified 6.0% CO_2_, 21% O_2_-containing incubator.

### Glucose Deprivation

Mixed cortical cultures and astrocyte cultures were deprived of glucose by thorough washing with and into BSS_0_. Glucose (final concentration = 10 mM) was immediately added to parallel cultures to serve as controls and added back to experimental conditions as indicated in each figure legend. In the MK-801 (R&D System, Rockford, IL) or LY367385 (R&D System, Rockford, IL) experiments, MK-801 was made as a 10 mM stock solution in H_2_O, and LY367385 was made as a 50 mM stock solution in 0.1 NaOH. The drugs or their corresponding vehicle were either given at the initiation of GD or spiked into desire groups at the times indicated in each figure legend.

### Measurement of Neuronal Cell Death

Cell death was quantitatively determined by the spectrophotometric measurement of lactate dehydrogenase (LDH) found in the cell culture medium as described in detail previously (Uliasz and Hewett, 2000). Data are expressed as a percentage of total neuronal LDH activity (defined as 100% cell death) determined by exposing parallel cultures to 200 µM of *N*-methyl-D-aspartate (NMDA) for 20 to 24 hr. As cultured astrocytes do not express NMDA receptors (Backus et al., 1989; Chan et al., 1990; B. Fogal and S.J. Hewett, unpublished data) and have previously been shown to survive up to 8 hr of GD (Jackman et al., 2012), LDH measurements can be used as a specific measure of neuron cell death.

### Quantitative Real-Time Polymerase Chain Reaction

Total RNA was isolated and first-strand cDNA synthesized as previously described (Uliasz et al., 2012). Quantitative real-time polymerase chain reaction (qPCR) was performed using mouse-specific primer pairs (Taqman Gene Expression Assays, Applied Biosystems: xCT [Mm00442530_m1] and β-actin [Mm01205647_g1]) per manufacturer’s instructions. Reactions were run in the Eppendorf Real-Time PCR System and relative quantification performed using the comparative cycle threshold method (ΔΔC_T_), where C_T_ values of xCT were normalized to β-actin C_T_ values from the same sample and then compared with a calibrator sample C_T_ value (control group, cultures kept in BSS_10_) to determine the relative fold increase in mRNA. β-actin C_T_ values are unaffected by GD.

### Radiolabeled L-Cystine and D-Aspartate Uptake

System x_c_^−^-specific ^14^C-L-cystine (PerkinElmer; Waltham, MA) and system X_AG_^−^-mediated ^3^H-D-aspartate (PerkinElmer; Waltham, MA) uptake was performed as previously described (Fogal et al., 2007). Cultures were washed into HCSS (3 × 750 µl) and allowed to equilibrate for 10 min (25℃). For *cystine uptake*, cells were incubated in HCSS containing 3 µM ^14^C-L-cystine (1 µCi/ml), 27 µM unlabeled cystine, 1 mM D-aspartate, and 0.5 mM acivicin (Enzo Life Sciences; Farmingdale, NY). D-aspartate and acivicin were included in the uptake buffer to block system X_AG_^−^ and γ-glutamyltranspeptidase, respectively. Uptake was terminated after 30 min by washing in ice-cold PBS (3 × 750 µl). For *D-aspartate uptake*, cells were incubated in HCSS containing 0.1 µCi/ml ^3^H-D-aspartate and 50 µM unlabeled D-aspartate (25℃) for 5 min and uptake terminated by washing cells with an ice-cold Na^+^-free choline stop buffer containing in mM: 116 choline chloride, 0.8 MgSO_4_, 1 KH_2_PO_4_, 10 HEPES, 5 KOH, 10 glucose, 0.9 CaCl_2_, and 5 nonradioactive D-aspartate.

Cells were lysed with warm 0.5% SDS and accumulated radioactivity estimated using a liquid scintillation counter. Readings of counts per minute from experimental conditions were corrected back to the original volume of lysate, and the picomoles of cystine and aspartate transported per minute were calculated as described (Fogal et al., 2007).

### Toxicity Bioassay

Purified astrocyte cultures were washed thoroughly into BSS containing or lacking 10 mM glucose. Sixty, 75, 90, or 105 min before the end of a 6-hr incubation period, glutamate—75 µM final well concentration—was spiked into the wells after which the supernatant was collected and transferred via 3/4 exchange to highly enriched murine neuronal cultures. Six hr later, neuronal cell death was quantified by measurement of the LDH released into the cell culture medium. Data are expressed as a percentage of total neuronal cell death facilitated by adding 200 µM glutamate to parallel neuronal cultures 1 day prior to experimentation. We demonstrated previously that the toxicity of the supernatant correlated with the glutamate-buffering capacity of astrocytes (Sen et al., 2011). Of note, glucose, to a final concentration of 10 mM, was added to all pooled media prior to its addition to neurons to prevent any confound associated with GD.

### Measurement of Glutamate

Two hundred microliters of supernatant harvested from the toxicity bioassay experiment (*vide supra*) were analyzed for glutamate concentration in the media via phenylisothiocyanate derivatization, high-performance liquid chromatography (HPLC) separation using a Hypersil-ODS reverse-phase column, and ultraviolet detection at 254 nm as described in (Fogal et al., 2007).

### Measurement of Adenosine Triphosphate

Adenosine triphosphate (ATP) levels were determined using the ATP Determination Kit (Molecular Probe™, Invitrogen; Eugene, OR) per manufacturer’s instruction. Culture wells were aspirated dry and the plate quick frozen on dry ice. After 5 min, plates were incubated at 37℃ for 10 min. One milliliter or 250 μl of a 1 × Cell Culture Lysis Reagent (Promega; Madison, WI) was added into each well of a 6-well or 24-well plate, respectively. Plates were gently shaken for 5 min. Cell lysates were collected, and intracellular ATP levels were measured and normalized to protein levels (BCA Assay; Pierce™, Pierce Biotechnology, Rockford, IL). When necessary, samples were diluted so that values fell within the linear range of the assay (0–1 µM).

### Excitotoxicity Assays

Mixed cortical cultures were deprived of glucose or incubated with glucose for the times indicated before being washed and exposed to either 25 µM NMDA or 100 µM glutamate in HCSS at room temperature. After 10 min, the exposure solution was washed away and replaced by MS supplemented with 10 µM glycine. The cells were transferred to a 37℃, normoxic (21%), 6% CO_2_ containing incubator overnight. Neuronal cell death was determined by the LDH assay, and data were expressed as a percentage of total neuronal LDH activity obtained by exposing parallel cultures to 200 µM of NMDA for 20 to 24 hr (defined as 100% neuronal cell death).

### Statistical Analysis

All statistical analyses were performed using GraphPad Prism (Version 6.0.1, GraphPad Software, Inc.) as described in each figure legend. As percentage data and normalized data are by nature nonnormally distributed, such data were first transformed via arcsin square root or −1 × log (Y), respectively, before analysis. Values of zero or less were set at 1 × 10^−20^ before transformation. In the case of qPCR, statistics were performed on the geometric means of 2^−ΔΔCT^ values. In all experiments, data were expressed as mean + *SEM*. Significance was assessed at *p* < .05.

## Results

**Figure 1.**
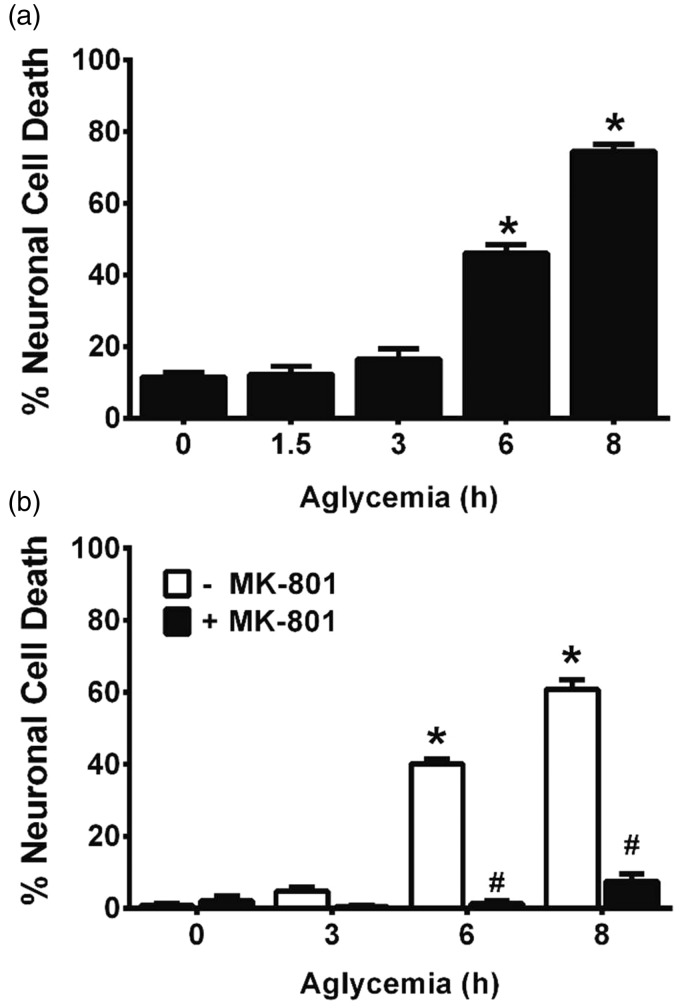
Aglycemic neuronal cell death is time-dependent and is blocked by NMDA receptor antagonism. (a) Mixed cortical cultures were washed into BSS containing (0 hr) or lacking 10 mM glucose for the times indicated, after which neuronal cell death was determined via measurement of LDH release. An asterisk (*) represents values significantly different from control (0 hr) as assessed by one-way ANOVA followed by Dunnett’s post hoc test (*n* = 8–10 cultures from three separate experiments). (b) Mixed cortical cultures were washed into BSS lacking or containing 10 mM glucose (0 hr) ± MK-801 (10 µM). Neuronal cell death was determined via measurement of LDH release. An asterisk (*) denotes a significant within-group difference, whereas a pound sign (#) denotes a significant between-group difference as determined by two-way ANOVA followed by Bonferroni’s post hoc test for multiple comparisons (*n* = 6 cultures from three separate experiments). NMDA = *N*-methyl-D-aspartate; BSS = balanced salt solution; LDH = lactate dehydrogenase; ANOVA = analysis of variance.

**Figure 2.**
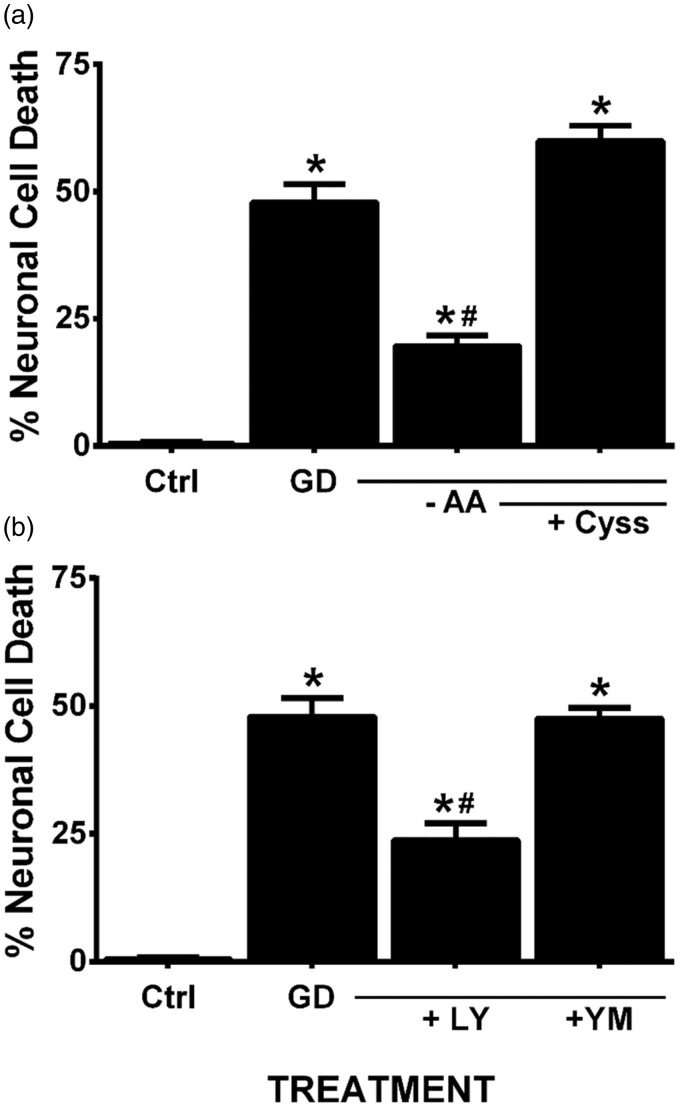
System x_c_^−^ contributes to aglycemic neuronal cell death. (a) Mixed cortical cultures washed into BSS_0_ containing (GD) or lacking (-AA) MEM amino acids save for supplementation with 100 µM L-cystine (+ Cyss). (b) Mixed cultures were deprived of glucose (GD) in the absence or presence of the dual system x_c_^−^/mGluR1 inhibitor, LY367385 (LY; 50 µM) or the selective mGluR1 antagonist, YM298190 (YM; 10 µM). Neuronal cell death was determined 8 hr later via measurement of LDH release. An asterisk (*) equals values different from control (cultures in BSS_10_), while a pound sign (#) represents a significant diminution from GD-induced injury as determined by one-way ANOVA followed by Bonferroni’s post hoc test for multiple comparisons (*n* = 10–11 from three independent experiments). GD = glucose deprivation; LDH = lactate dehydrogenase; BSS = balanced salt solution; ANOVA = analysis of variance.

In confirmation of prior literature from our own laboratory as well as that from others, we demonstrate that removal of glucose from the cell culture medium of mixed neuronal/astrocyte cortical cell cultures results in a time-dependent (Figure 1(a)) and NMDA receptor-mediated excitotoxic neuronal cell death (Figure 1(b); Monyer and Choi, 1988; Monyer et al., 1989; Jackman et al., 2012). That system x_c_^−^ activity contributes to death is illustrated by the fact that removal of amino acids (Figure 2(a))—specifically cystine (Jackman et al., 2012)—from the glucose-free medium reduced aglycemic neuronal injury, whereas addition of cystine back to the medium lacking amino acids results in significant neuronal cell death (Figure 2(a)). Furthermore, addition of the dual mGluR1/system x_c_^−^ antagonist LY367385 ameliorates aglycemic neuronal cell death, while the mGluR1-specific antagonist, YM 298198, shows no protection (Figure 2(b); Jackman et al., 2012). While neurons and astrocytes both express functional system x_c_^−^ in our culture system (Jackman et al., 2010), we recently described astrocytic system x_c_^−^ as the main source of glutamate required for the initiation of neuronal injury following GD *in vitro* (Jackman et al., 2012).

**Figure 3.**
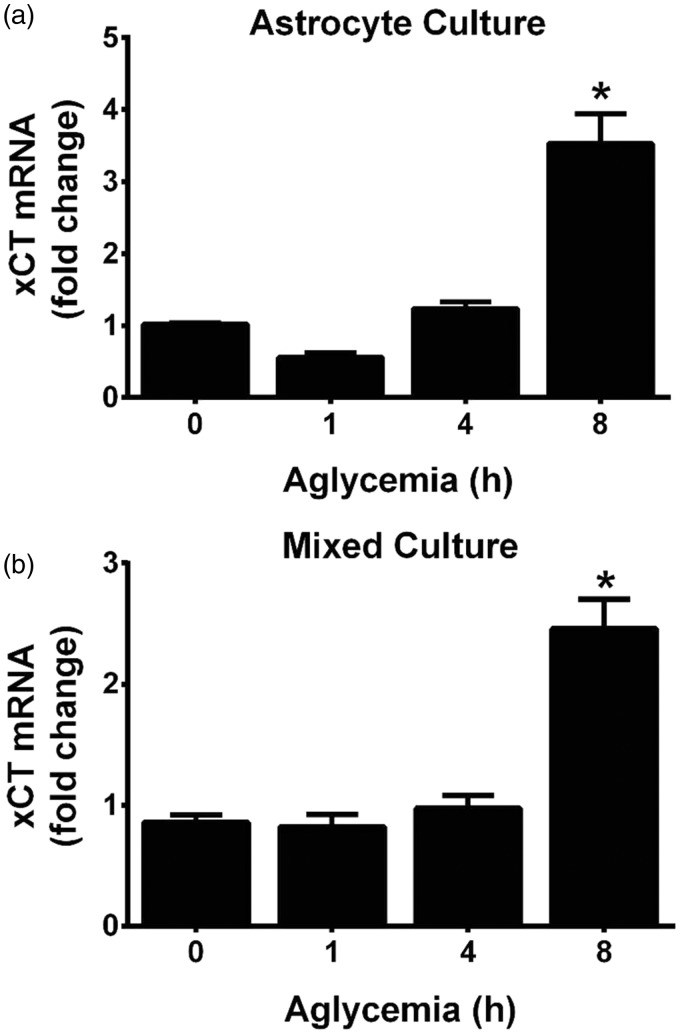
Aglycemia regulates xCT mRNA levels in a time-dependent manner. (a) Purified astrocyte cultures or (b) mixed cortical cultures containing both astrocytes and neurons were deprived of glucose for the times indicated and changes in xCT mRNA assessed via qPCR. An asterisks (*) represent values that are significantly different from 0 hr (i.e., glucose-containing control) as determined by one-way ANOVA followed by a Dunnett’s post hoc test for multiple comparison (*n* = 4 from four separate experiments each). qPCR = quantitative real-time polymerase chain reaction; ANOVA = analysis of variance.

**Figure 4.**
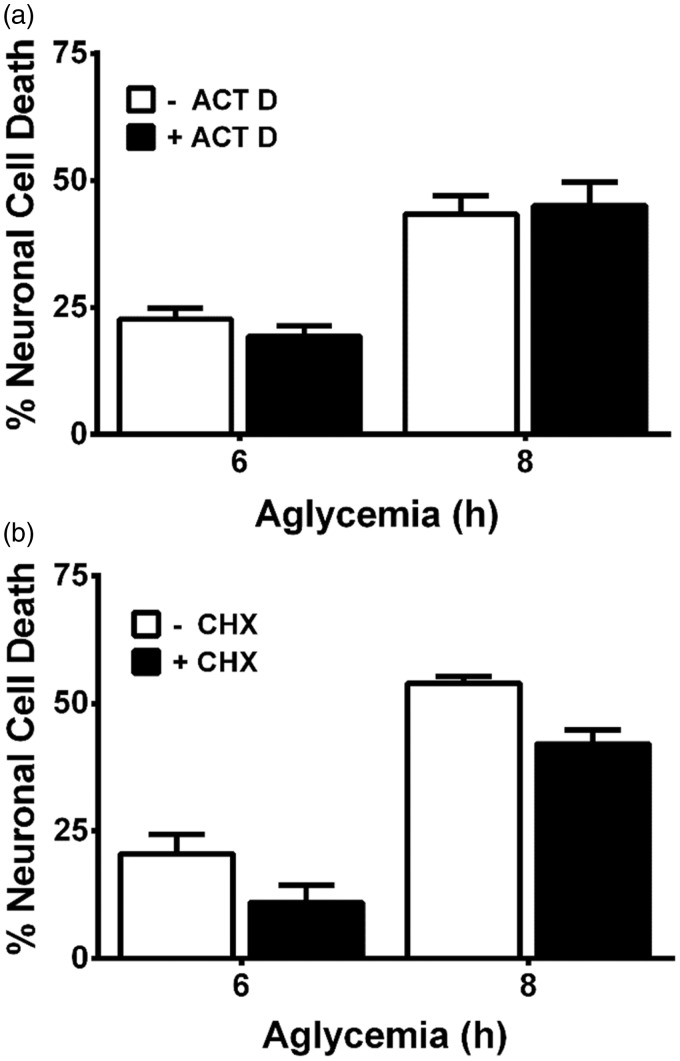
Macromolecular synthesis inhibitors do not prevent aglycemic neuronal cell death. Mixed cortical cultures were rendered aglycemic in the presence or absence of (a) the transcriptional inhibitor actinomycin D (ACT D; 10 µg/ml; *n* = 6–10 from five separate experiments) or (b) the protein synthesis inhibitor cycloheximide (CHX; 1 µg/ml; *n* = 12 from three separate experiments). Six and 8 hr later, the amount of neuronal cell death was assessed. (a, b) Two-way ANOVA revealed no significant between-group differences in either paradigm. ANOVA = analysis of variance.

Cellular stress results in transcriptional adaptive increases in the substrate-specific light chain of system x_c_^−^, xCT, and a subsequent increase in cystine uptake (Bannai, 1984a; Ishii et al., 1992; Miura et al., 1992; Ishii et al., 2000; Sasaki et al., 2002; Sato et al., 2004). Hence, whether xCT mRNA levels was altered following aglycemia in pure astrocyte cultures or in mixed cortical cultures containing both astrocytes and neurons was ascertained using quantitative PCR analysis. In both astrocyte and mixed cortical cell cultures, a significant increase in xCT mRNA occurs following 8 hr of GD but not earlier (Figure 3(a) and (b)). Because significant and nearly maximal cell death occurs by this time point (Figure 1), we find it unlikely that a change in system x_c_^−^ expression underlies its neurotoxic effect. In keeping with this, actinomycin D (10 µg/ml), when given at the onset of GD, fails to prevent aglycemic neuronal cell death (Figure 4(a)), demonstrating no requirement for transcription. Death is similarly unaffected by cycloheximide (1 µg/ml), indicating no need for protein synthesis (Figure 4(b)).

**Figure 5.**
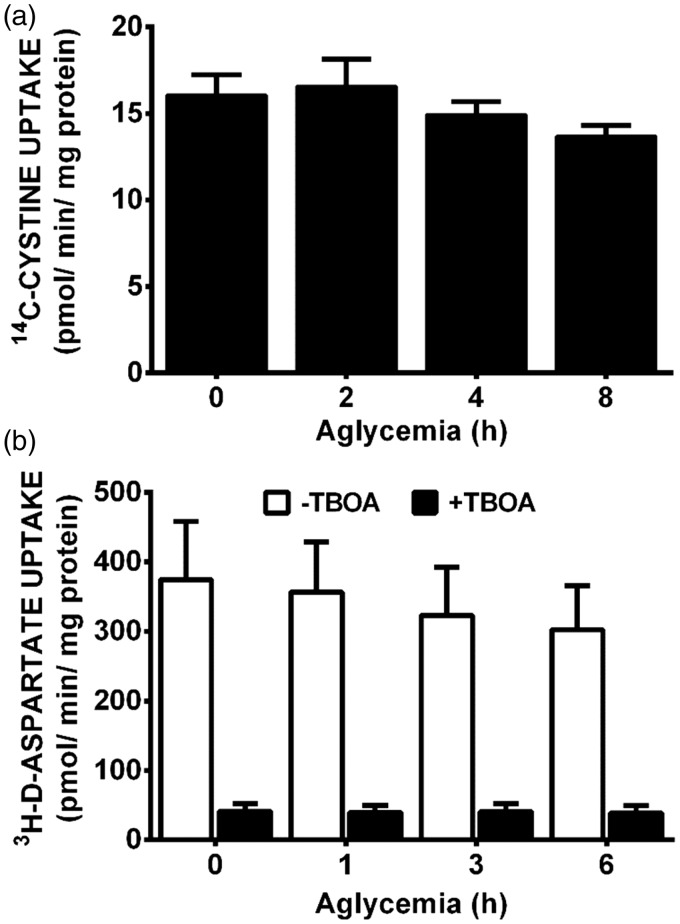
Aglycemia does not significantly alter cystine or aspartate uptake. (a) Mixed cortical cultures were deprived of glucose for the times indicated, then incubated with buffer containing ^14^C-L-cystine for 30 min to measure system x_c_^−^ activity. Data are expressed as mean +* SEM* ^14^C-L-cystine uptake in pmol/min/mg protein. Aglycemia had no significant effect on radiolabeled cystine uptake as determined by one-way ANOVA followed by a Dunnett’s post hoc test (*n* = 6 from two separate experiments). (b) Mixed cortical cultures were deprived of glucose for the times indicated, then ^3^H-D-aspartate was added for 5 min to measure EAAT activity. D,L-threo-β-benzyloxyaspartate (500 µM) was used to confirm uptake via EAATs (*n* = 6 from five separate experiments). Data are expressed as mean + *SEM* ^3^H-D-aspartate uptake in pmol/min/mg protein. (a, b) ANOVA revealed no significant within-group differences (compared with 0 hr) in each paradigm. TBOA = D,L-threo-β-benzyloxyaspartatemethionine; EAAT = Excitatory Amino Acid Transporter; ANOVA = analysis of variance.

**Figure 6.**
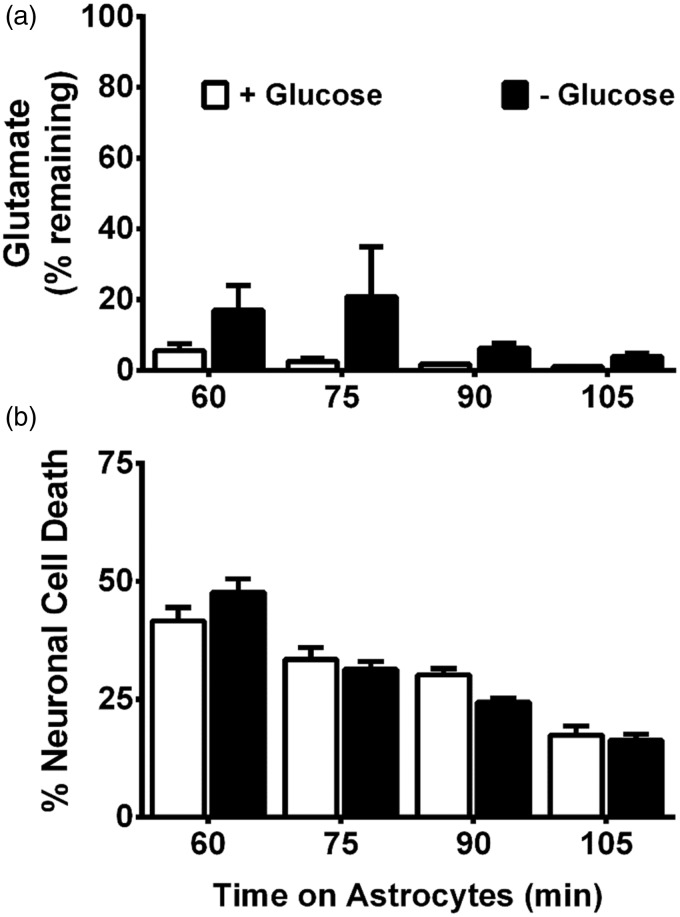
Aglycemic astrocytes are still capable of glutamate uptake. Purified astrocyte cultures were glucose deprived (− Glucose) or washed into BSS containing glucose (+Glucose; 10 mM) for 6 hr before glutamate (final concentration = 75 µM) was added to cultures. At the times indicated after addition, bathing medium was removed and like groups pooled. (a) HPLC analysis was used to measure remaining glutamate in an aliquot of the pooled media. Data are expressed as percentage of initial glutamate concentration (75 µM; *n* = 3–6 from three separate experiments). (b) The pooled medium was then added to pure neuronal cultures (2/3 exchange). Six hr later, neuronal cell death was assessed. (*n* = 10 from five separate experiments). (a, b) No significant between-group differences were found using two-way ANOVA. HPLC = high-performance liquid chromatography; BSS = balanced salt solution; ANOVA = analysis of variance.

To ascertain whether system x_c_^−^ activity was enhanced by GD, we measured ^14^C-L-cystine uptake in the mixed cortical cell cultures. No change in activity is noted over the time-course of GD studied (Figure 5(a)), suggesting increased glutamate extrusion via system x_c_^−^ does not underlie its neurotoxic effect. As maintenance of extracellular glutamate concentrations involves control of release as well as uptake, we next investigated whether aglycemic-mediated alterations in glutamate removal occurred. We find no significant difference in ^3^H-D-aspartate uptake—a measure of EAAT activity as verified by the ability of D,L-threo-β-benzyloxyaspartate to completely suppress the uptake—between control mixed cultures and those that were deprived of glucose (Figure 5(b)). Also, glucose-deprived astrocytes retain the ability to remove exogenously added glutamate just as effectively as control astrocytes (Figure 6(a)). Finally, glutamate-containing media removed from glucose-deprived astrocytes is equally as toxic to pure neuronal cultures as that from glucose-containing medium at every time point assessed (Figure 6(b)). Therefore, overstimulation of neuronal excitatory amino receptors via enhancement of the extracellular glutamate concentration cannot account for the excitotoxic insult mediated by system x_c_^−^ under aglycemic conditions.

**Figure 7.**
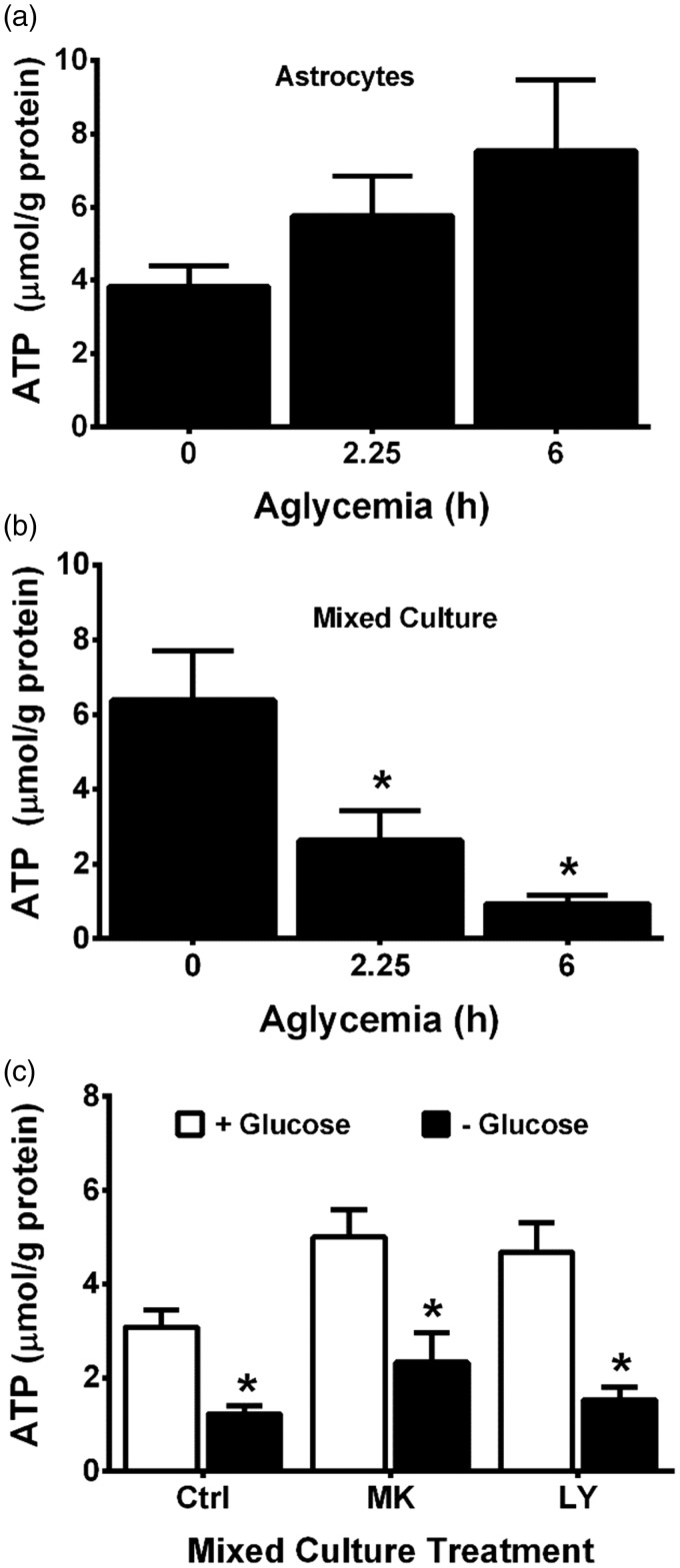
Aglycemia decreases ATP levels in cortical neurons but not astrocytes. (a) Purified cortical astrocyte cultures (*n* = 7–8 from two independent experiments) or (b) mixed cortical cultures (*n* = 3–4 from one experiment) were placed in medium containing glucose for 6 hr (0) or deprived of glucose for the times indicated, after which ATP levels were measured as described in methods. (c) Mixed cortical cultures were washed into BSS lacking (− Glucose, black bars) or containing 10 mM glucose (+Glucose, white bars) ± MK-801 (MK; 10 µM) or LY367385 (LY; 50 µM). Six hr later, ATP levels were measured (*n* = 6 from two independent experiments). Data are expressed as mean + *SEM*. Individual values are normalized to each well’s protein levels. (a, b) An asterisk (*) indicates a significant difference from 0 hr as determined by one-way ANOVA followed by a Dunnett’s post hoc test for multiple comparison. (c) An asterisk indicates a significant between-group difference as determined by two-way ANOVA followed by Bonferroni’s post hoc test for multiple comparisons. ATP = adenosine triphosphate; BSS = balanced salt solution; ANOVA = analysis of variance.

Consequently, we set out to test whether the *weak excitotoxic hypothesis* (Albin and Greenamyre, 1992)—which posits that bioenergetic deficiencies enhance the toxic effect of glutamate—might explain the results described earlier. Following GD *in vitro*, rapid ATP depletion has been reported to occur in neurons with astrocytes being more resistant (Choi et al., 2008). Likewise, we find that aglycemic astrocytes maintain cellular ATP levels (Figure 7(a)), whereas the concentration of ATP in mixed cortical cultures is significantly and severely depressed when glucose was removed from the media (Figure 7(b)). These changes are not simply due to neuronal loss from the mixed cultures because the addition of MK-801 and LY367385, which protects the neurons from aglycemia-induced death (Figures 1(b) and 2(b)), does not rescue the culture ATP levels (Figure 7(c)).

**Figure 8.**
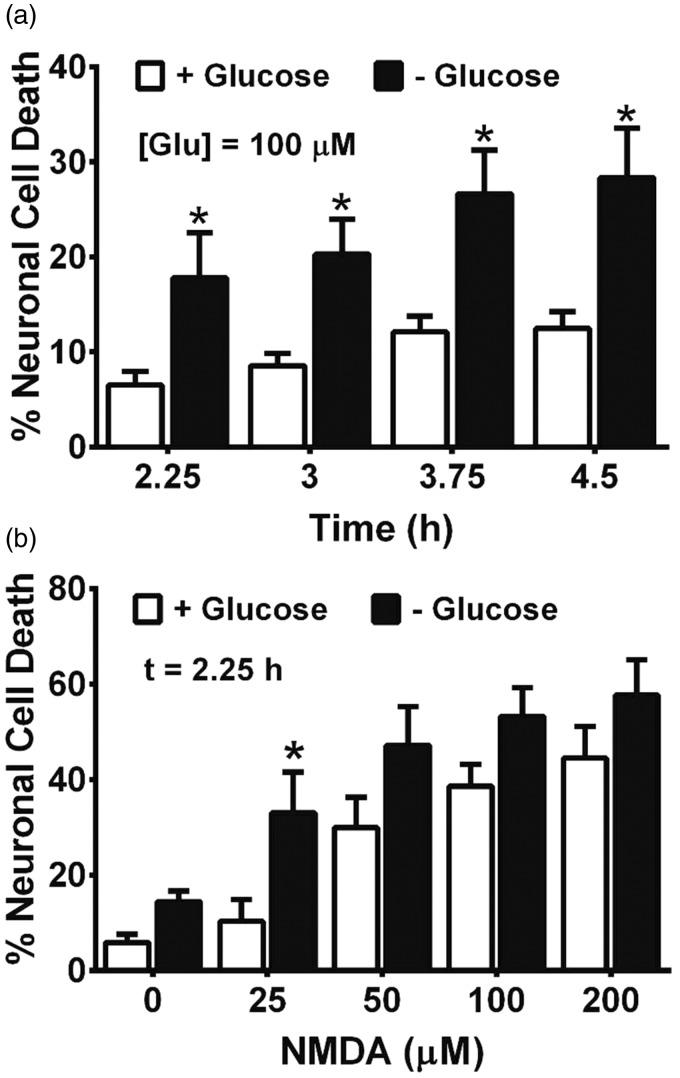
Glucose-deprived mixed cultures are more susceptible to the toxic effects of glutamate and NMDA. Mixed cortical cultures (a) were deprived of glucose for the times indicated followed by exposure to 100 µM glutamate (*n* = 6 from three separate experiments) or (b) were deprived of glucose for 2.25 hr followed by exposure to increasing concentrations of NMDA (*n* = 6 from three separate experiments). Ten min later, cultures were washed free of glutamate or NMDA and placed back into a glucose-containing medium. The percentage of neuronal cell death was assessed 20 to 24 hr later by measurement of LDH. An asterisk (*) indicates a significant between-group difference assessed by two-way ANOVA followed by a Bonferroni’s post hoc test for multiple comparison. NMDA = *N*-methyl-D-aspartate; LDH = lactate dehydrogenase; ANOVA = analysis of variance.

To investigate whether aglycemic cortical neurons would show an enhanced susceptibility to excitotoxic insult, we deprived mixed cultures of glucose for increasing periods of time (2.5 to 4.5 hr) after which glutamate (100 µM final well concentration) was added for 10 min. Compared with cells maintained in glucose-containing medium, results show that glutamate kills significantly more neurons when the cultures are glucose deprived at every time point tested (Figure 8(a)). Likewise, neurons in glucose-deprived cultures are more susceptible to death than control cultures when exposed to equimolar concentrations of NMDA (Figure 8(b)).

**Figure 9.**
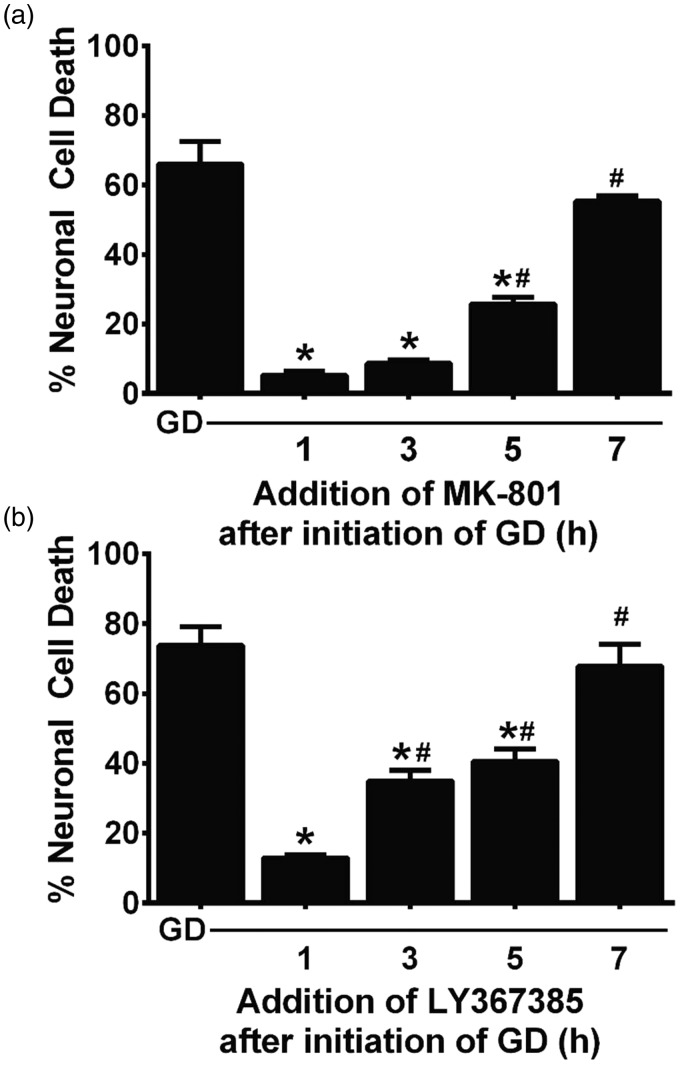
Delayed pharmacological rescue of aglycemic neuronal injury in mixed cortical cultures. Mixed cortical cultures were deprived of glucose for a total 8 hr (GD). (a) MK-801 (10 µM) or (b) LY367385 (50 µM) were added at the times indicated after the initiation of GD. The percentage of neuronal cell death was assessed via the LDH assay. An asterisks (*) indicates a significant difference from GD, and a pound sign (#) indicates a significant difference from 1 hr determined by one-way ANOVA followed by Dunnett’s post hoc test for multiple comparison (*n* = 6 from two experiments). GD = glucose deprivation; LDH = lactate dehydrogenase; ANOVA = analysis of variance.

Finally, to study the optimal therapeutic time window for rescue, mixed cortical cultures were deprived of glucose for a total of 8 hr and either MK-801, to block NMDA receptors (Figure 9(a)), or LY367385 (Figure 9(b)), to mitigate glutamate release from system x_c_^−^, was added to parallel cultures at 1- to 2-hr intervals postexperimental onset. The percentage of the dying neuronal population that could be rescued depended on the time interval between washout of glucose and addition of the pharmacological agents. When compared with 8 hr of GD alone, MK-801 is able to completely block aglycemic neuronal injury when given up to 3 hr after the start of GD, and it is still significantly effective when addition is delayed for 5 hr (Figure 9(a)). In contrast, maximal protection for LY367385 occurs at 1 hr, though significant protection is also demonstrated up to 5 hr (Figure 9(b)). No protection is observed with either compound if its administration is delayed by 7 hr (Figure 9(a) and (b)).

## Discussion

Symptomatic hypoglycemia, resulting in brain dysfunction ranging from mild behavioral impairments to coma (Wilson, 1983), is the principal problem associated with tight glucose regulation in patients undergoing insulin therapy (Amiel et al., 1987; Group, 1991; Cryer, 2001; Griesdale et al., 2009). The idea that the underlying cause of severe neurological complications that follow a dangerous drop in blood glucose levels is not directly due to substrate deprivation-induced energy failure has been recognized since the early 1970s (for review, see Auer, 1986). Instead, the neuronal cell death that ensues occurs via excitotoxicity (Wieloch, 1985; Monyer and Choi, 1988; Monyer et al., 1989; Papagapiou and Auer, 1990; Nellgard and Wieloch, 1992; Tasker et al., 1992; Ichord et al., 2001; Jackman et al., 2012), the process of which is most commonly ascribed to overexcitation of neuronal glutamate receptors (Bo et al., 1994). While multiple sources of glutamate could contribute to aglycemic neuron death (Danbolt, 2001), *in vivo and in vitro* studies indicate a contributory role for neuronal exocytotic release of glutamate (Wieloch et al., 1985; Monyer et al., 1989; Jackman et al., 2012) as well as an obligate requirement for astrocytic system x_c_^−^ (Jackman et al., 2012). The mechanism by which astrocytic system x_c_^−^ contributes to aglycemic neuronal injury has been explored in this study.

Using a mixed cortical cell culture system *in vitro*, we demonstrated previously that enhancement of astrocyte system x_c_^−^ (cystine/glutamate antiporter) expression and activity facilitated by interleukin-1β contributed to the development and progression of hypoxic neuronal injury—a model of the ischemic penumbra (Fogal et al., 2007; Jackman et al., 2010). While the enhanced extrusion of glutamate from astrocytic system x_c_^−^ activity was not deleterious in and of itself, we found that under hypoxic conditions, astrocyte glutamate uptake was also compromised, thus leading to glutamate buildup (Fogal et al., 2007). Thus, we speculated that similar mechanisms might be in play in our hypoglycemia model described herein.

With respect to upregulation of system x_c_^−^, we did find that xCT mRNA expression in astrocytes and mixed cultures was increased in a time-dependent manner following GD. However, in agreement with our previous study (Jackman et al., 2012), a statistically significant increase did not occur in either culture system until 8 hr following removal of glucose from the medium (Figure 3). Given that much, if not all, of the death has already occurred by this time point, it seems unlikely that a change in system x_c_^−^ expression is necessary to mediate the deleterious effects of system x_c_^−^ under aglycemic conditions. Indeed, actinomycin D or cycloheximide—used at concentrations shown previously by us to effectively decrease the transcriptional (10 µg/ml) and translational (1 µg/ml) increases in astrocyte xCT facilitated by interleukin-1β, respectively (Jackman et al., 2010)—failed to ameliorate aglycemic neuronal injury (Figure 4). However, because system x_c_^−^ transport can be enhanced via phosphorylation of putative protein kinase A sites in xCT (Baker et al., 2002b) and changes in protein trafficking could lead to changes in xCT protein insertion in the membrane (Chase et al., 2013; Cramer and Chase, 2013; Ladd et al., 2014), we also investigated the activity of system x_c_^−^ activity using a radiolabeled cystine uptake assay. Our results directly demonstrate that no change in system x_c_^−^ activity occurs following GD (Figure 5(a)).

Following release, the concentration of glutamate in the extracellular space is carefully maintained at subtoxic levels by its rapid reuptake. We initially surmised that glucose-deprived astrocytes might show a diminution in their ability to remove glutamate from the extracellular space, thus leading to a toxic buildup. However, using three different assays—^3^H-D-asparte uptake (Figure 5(b)), direct measurement of residual glutamate levels in astrocyte media via HPLC following exogenous addition of glutamate (Figure 6(a)), and a toxicity bioassay (Figure 6(b)) developed by our laboratory and validated for its ability to accurately reflect glutamate uptake into astrocytes (Sen et al., 2011)—we demonstrate no change in the ability of glucose-deprived astrocytes alone or astrocytes contained in mixed culture to remove glutamate from the extracellular environment. While this might initially seem surprising, others have shown that there were no changes in astrocytic glutamate uptake in astrocytes for up to 2 hr of GD (Bakken et al., 1998) and only a 20% loss after 24 hr (Swanson and Benington, 1996). Indeed, unlike neurons (Auer et al., 1984; Monyer and Choi, 1988; Monyer et al., 1989; Goldberg and Choi, 1993), astrocytes have been demonstrated to be more resistant (Monyer and Choi, 1988; Monyer et al., 1989; Goldberg and Choi, 1993; Lyons and Kettenmann, 1998) to the ATP-depleting effects of glucose starvation by virtue of their ability to use glycogen stores (Cataldo and Broadwell, 1986; Swanson et al., 1990) that can be metabolized to meet their metabolic needs (Swanson et al., 1990; Erecinska and Silver, 1994; Dienel and Cruz, 2006; Walls et al., 2009). In addition, astrocytes can metabolize glutamate via the TCA cycle to provide energy (Yu et al., 1982; Yu et al., 1984; McKenna et al., 1996; McKenna, 2007), especially when glucose levels are low (Bakken et al., 1998). Indeed, we found that astrocytes are able to fully maintain their ATP levels under the conditions of GD used in this study (Figure 7(a)). Thus, the loss in mixed culture is then likely due to changes in neuronal ATP production (Figure 7(b)). These results are in good agreement with what has been reported previously (Choi et al., 2008).

It is important to point out that neither enhanced release of glutamate nor its diminished uptake may be needed to facilitate excitotoxic neuronal cell death under aglycemic conditions, as Novelli et al. demonstrated nearly 20 years ago that glutamate concentrations needed to kill energy-deprived cerebellar neurons are far less than those required to kill healthy neurons (Novelli et al., 1988; Henneberry et al., 1989). These observations, among others, led to the development of the weak excitotoxin hypothesis (Albin and Greenamyre, 1992), which postulates that a reduction in cellular ATP leads to an impairment in Na^+^/K^+^ ATPase activity resulting in a slight membrane depolarization (Calabresi et al., 1997) that ultimately allows for voltage-dependent NMDA receptors to be more easily activated (Henneberry et al., 1989; Beal, 1992). Indeed, the reduction in neuronal ATP correlated with the toxicity of glutamate (Henneberry et al., 1989) and poisoning of the Na^+^ pump with ouabain replicated what was found when glucose was removed from the medium (Novelli et al., 1988). In keeping with this hypothesis, we found that glucose-deprived cultures showed a two- to threefold enhancement in neuronal cell death when exposed to 100µM glutamate at nearly every time point of GD analyzed when compared with their glucose-containing controls (Figure 8(a)). Similar results were seen when NMDA was used (Figure 8(b)). As one might expect, a longer therapeutic window of opportunity, at least when it comes to maximal or essentially full protection, was achieved by blocking NMDA receptors when compared with blocking system x_c_^−^ itself, although both drugs used showed significant protection up to a remarkable 5 hr following removal of glucose (Figure 9).

Finally, the fact that system x_c_^−^ contributes to injury might seem paradoxical given its importance in the biosynthesis of the antioxidant molecule glutathione (Watanabe and Bannai, 1987; Bannai et al., 1989; Miura et al., 1992; Sato et al., 1995; Bridges et al., 2001; Dun et al., 2006; Lewerenz et al., 2009). Ironically, while this pathway allows the CNS to rapidly upregulate glutathione production in response to cellular stress, we and others demonstrate its potential to exacerbate CNS pathology both *in vitro* and *in vivo* (for reviews, see Bridges et al., 2012; Lewerenz et al., 2013; Massie et al., 2015). As such, it is becoming increasingly clear that the consequences of system x_c_^−^ activity are complex and context-dependent. Hence, system x_c_^−^ activity can either be beneficial (Tanaka et al., 1999; Shih et al., 2003; Jakel et al., 2007; He et al., 2015) or can contribute to pathophysiology (Piani and Fontana, 1994; Ye et al., 1999; Barger and Basile, 2001; Qin et al., 2006; Fogal et al., 2007; Savaskan et al., 2008; Sontheimer, 2008; Jackman et al., 2010; Massie et al., 2011; Jackman et al., 2012), the ultimate effect being dependent on the environmental and cellular milieu.

In sum, we find that aglycemic neuronal cell death does not result from changes in astrocyte system x_c_^−^ expression or activity or from impairment of glutamate removal. Instead, our results are consistent with the weak excitotoxic hypothesis (Albin and Greenamyre, 1992), suggesting that the bioenergetic deficiencies, which are present in our mixed cultures, enhance the toxic effect of physiological levels of glutamate released from astrocyte system x_c_^−^. Whether system x_c_^−^ plays a similar role in hypoglycemic neurodegeneration *in vivo* requires further experimentation.
